# Distinctively different predictors for long‐term outcomes between responders and nonresponders who underwent cardiac resynchronization therapy

**DOI:** 10.1002/joa3.12447

**Published:** 2020-11-10

**Authors:** Kunio Yufu, Ichitaro Abe, Hidekazu Kondo, Shotaro Saito, Akira Fukui, Norihiro Okada, Hidefumi Akioka, Tetsuji Shinohara, Yasushi Teshima, Mikiko Nakagawa, Naohiko Takahashi

**Affiliations:** ^1^ Department of Cardiology and Clinical Examination Faculty of Medicine Oita University Oita Japan

**Keywords:** cardiac resynchronization therapy, heart failure, long‐term outcome, renal function, responder

## Abstract

**Background:**

It is common to develop heart failure (HF) events even in respondents to cardiac resynchronization therapy (CRT) during a long‐term observation period. We investigated the predictors for long‐term outcome in responders in comparison with nonresponders in patients diagnosed with HF along with implanted CRT.

**Methods:**

We enrolled 133 consecutive patients (mean age, 70 ± 10 years; 72 males) implanted with CRT from April 2010 to July 2019. Accurate follow‐up information (mean follow‐up period, 983 ± 801 days) was obtained from 66 responders and 53 nonresponders.

**Results:**

Kaplan‐Meier event‐free curves showed that major adverse cerebral and cardiovascular event (MACCE)‐free ratio was significantly lower as the stage of renal function progresses (log rank, 19.5; *P* < .0001). The baseline estimated glomerular filtration rate (e‐GFR) before CRT was not significantly different between nonresponders and responders. The e‐GFR after judgment of CRT response was lower in patients with MACCEs than those without. Cox proportional hazards regression analysis revealed that low baseline e‐GFR before CRT and after judgment of CRT response was closely related with MACCEs in responders, but not in nonresponders. The survival rate in responders without MACCEs assessed using Kaplan‐Meier analysis was significantly larger in the preserved e‐GFR (baseline value before CRT, >44 mL/min/1.73 m^2^) group than in the depressed group (log rank, 20.29; *P* < .0001).

**Conclusion:**

We demonstrate that the factors for MACCEs during long follow‐up periods were distinctively different between responders and nonresponders. Patients with depressed e‐GFRs are suggested to have poor prognosis even if they are responders to CRT.

## Graphical abstract

Our results suggest that the factors for MACCE during long follow‐up period were distinctively different between responders and nonresponders. Patients with depressed e‐GFR are suggested to have poor prognosis even if they are responders to CRT.

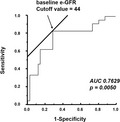

## 1 INTRODUCTION

For patients with severe heart failure (HF), there is no doubt that cardiac resynchronization therapy (CRT) can be the beneficial method on improving cardiac function, subjective symptoms, exercise capacity, and outcome.^1^, ^2^, ^3^, ^4^ Responders to CRT demonstrating reverse left ventricular (LV) remodeling after 6 months show good clinical outcomes after CRT.^5^ However, it is common to develop HF events even in responders through long life cycle. In a study, approximately about 30% or more of the responders became transient responders 2 years after CRT. The transient responders showed significantly greater hospitalization rates for HF than responders with persistent response.^6^ Nevertheless, it was not very clear about whether the factors predicting cerebral and cardiovascular events would be different between responders and nonresponders after a long‐term follow‐up.

Cardiorenal syndrome comprises clinical conditions where both cardiac and renal dysfunctions coexist. It results in impaired cardiac systolic function and glomerular filtration, which lead to cardiac volume decompensation.^7^ This study determines the factors predicting long‐term prognosis (cerebral and cardiovascular events) in responders in comparison with those in nonresponders with HF who underwent CRT.

## 2 MATERIALS AND METHODS

### 2.1 Patient selection

**FIGURE 1:**
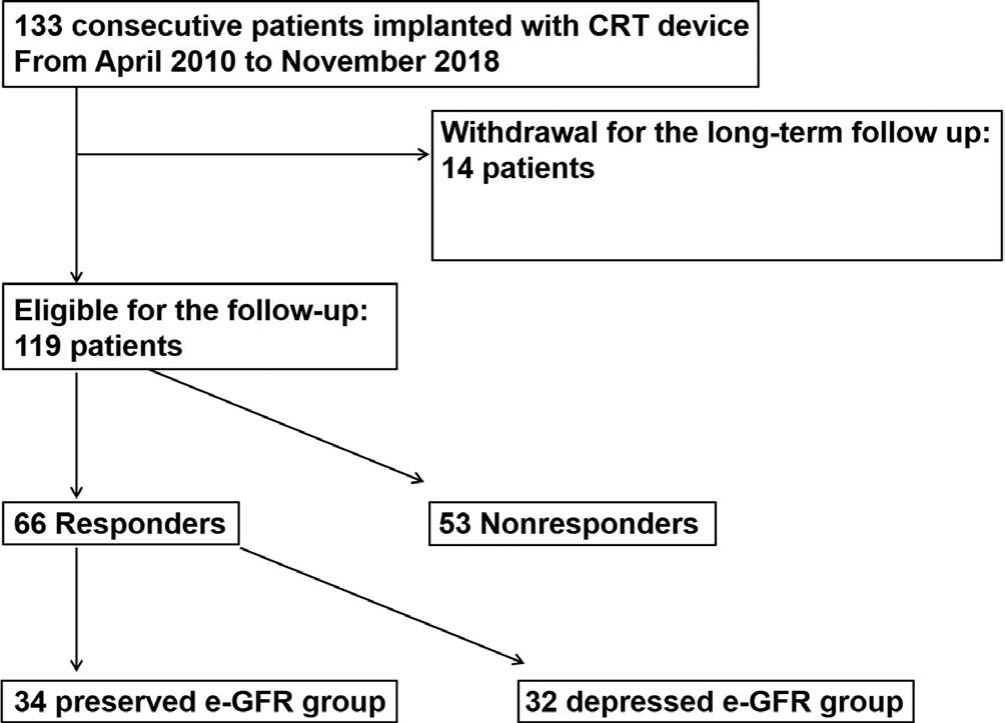
Flowchart of 133 consecutive patients undergoing CRT devices from April 2010 to November 2018. CRT, cardiac resynchronization therapy; estimated glomerular filtration rate (e‐GFR)

We identified and considered 133 patients with HF who had a CRT pacemaker implanted for the therapy of severe HF from April 2010 to July 2019. In this study, the indications for CRT adhere to the Japanese Circulation Society guidelines.^8^ Patients who lack long‐term follow‐up and those undergoing dialysis were omitted. A total of 119 patients were eligible and registered to the study (Figure 1). CRT responders were defined as patients with over 15% reduction in the left ventricular end‐systolic volume (LVESV).^9^ at the least 6 months after undergoing CRT operation. Brain natriuretic peptide (BNP) or pro‐BNP, and estimated glomerular filtration rate (e‐GFR) were evaluated before CRT as the baseline value. They were also evaluated just after judgment of CRT response.

Patients who had readmitted due to HF, who had worsening of the NYHA class on the final follow‐up observation, who had continuous moderate‐to‐severe deterioration on the clinical composite score^10^ at the final medical examination, who lastingly stopped biventricular pacing due to worsening HF, or who died within 6 months after CRT were assigned to nonresponders.^9^

This research was approved by the ethics review board of Oita University, and we received informed consent from whole patients. Our study was directed consistent with the principles expressed in the Declaration of Helsinki of the World Medical Association.

### 2.2 Echocardiographic assessment

We performed M‐mode, 2D, and Doppler echocardiographic measurements by a 1.5‐4.0 MHz transducer at a fitting depth on apical and parasternal views. LVESV, left ventricular end‐diastolic volume (LVEDV), and LV ejection fraction (LVEF) were evaluated using echocardiography by modified biplane Simpson's rule.

### 2.3 Follow‐up

All 119 patients performed the complete examination about CRT, and the status of clinical function was checked every 4‐6 months after operation. Exact follow‐up data for 983 ± 801 days were obtained. The endpoint was prespecified as the occurrence of major adverse cerebral and cardiovascular events (MACCEs), which contained stroke, nonfatal myocardial infarction, cardiovascular death, percutaneous coronary intervention or coronary artery bypass graft, implantable cardioverter‐defibrillator treatment for fatal arrhythmia, and hospitalization due to congestive HF. Among the aforementioned events, the statistical analysis only considered the first event.

### 2.4 Statistical Analysis

Data were shown in mean ± SD. The analysis of variance was used for continuous variables, and the Chi‐squared test was used for categorical variables. The Student's *t* test was used to analyze the difference between groups. Univariate Cox proportional hazard regression analyses were done to distinguish the factors for prediction of MACCEs. The risk model was applied and gender, age, heart rates, blood pressures, etiologies of cardiomyopathy, QRS durations, LVEF, LVESV, LVEDV, BNP, and e‐GFR were contained in the risk factors. The results are given as hazard ratios with 95% confidence intervals. A *P* value <0.1 was considered statistically substantial. Multiple Cox regression analysis was performed using values which had significance in a univariate analysis. Kaplan‐Meier survival analysis was performed to associate MACCE‐free ratio among the preserved and depressed e‐GFR groups. All analyses were calculated by JMP (version 13.2.0; SAS Institute, Cary, NC, USA).

## 3 RESULTS

### 3.1 Clinical characteristics

Accurate follow‐up information (mean follow‐up duration, 983 ± 801 days) was obtained from 66 responders and 53 nonresponders. Table 1 showed baseline characteristics of the responders and the nonresponders. Significant difference was not obtained regarding gender, age, QRS duration, heart rate, blood pressures, echocardiographic measurements, and values of blood samplings between the responders and the nonresponders. Regarding primary HF etiology, the number of patients with dilated cardiomyopathy was larger in the responders compared to the nonresponders. There was no significant difference regarding the prevalence of atrial fibrillation between nonresponders and responders (7.6% vs 16.7%, *P* = .13), and between patients with MACCEs and without (15.8% vs 9.7%, *P* = .31), respectively.

**TABLE 1:** Baseline clinical characteristics

	Nonresponders	Responders	*P* value
	(n = 53)	(n = 66)	
Age (y)	70 ± 10.2	69 ± 9.8	.59
Gender (Female/male)	18/ 35	28/ 38	.35
Ischemic cardiomyopathy	11	10	.43
Dilated cardiomyopathy	17	36	.014*
Cardiac sarcoidosis	12	9	.20
Pacing induced cardiomyopathy	1	5	.14
Cardiac amyloidosis	3	1	.21
Valvular disease	6	2	.07
Others	3	3	.78
Atrial fibrillation	4 (7.6%)	11 (16.7%)	.13
QRS duration (ms)	161 ± 27	168 ± 24	.11
SBP (mmHg）	109 ± 19	114 ± 19	.12
DBP (mmHg)	65 ± 11	66 ± 11	.61
Heart rate (beats/min)	65 ± 16	65 ± 12	.95
LVEF (%)	32 ± 8.6	30 ± 8.4	.33
End‐diastolic volume (ml)	164 ± 63	162 ± 61	.88
End‐systolic volume (ml)	113 ± 50	116 ± 51	.78
Pro‐BNP (pg/ml) before CRT	5960 ± 8013 (n = 8)	4557 ± 5546 (n = 4)	.76

Abbreviations: before CRT, baseline value before CRT; BNP, brain natriuretic peptide; CRT, cardiac resynchronization therapy; DBP, diastolic blood pressure; e‐GFR, estimated glomerular filtration rate; LVEF, left ventricular ejection fraction; SBP, systolic blood pressure.

* *P* < .05.

### 3.2 The chronological changes of e‐GFR and BNP and the occurrence of MACCE

The rates of responder to CRT among the groups based on stages of CKD^11^ before CRT implantation were not significant (*P* = .601), as follows. The rates of responder to CRT were 63.6% in the patients with e‐GFR ≥ 60 mL/min/1.73 m^2^ (n = 22), 52.1% in the patients with e‐GFR <60 and ≥30 ml/min/1.73 m^2^ (n = 73), and 58.3% in the patients with e‐GFR < 30 mL/min/1.73 m^2^ (n = 24).

**FIGURE 2:**
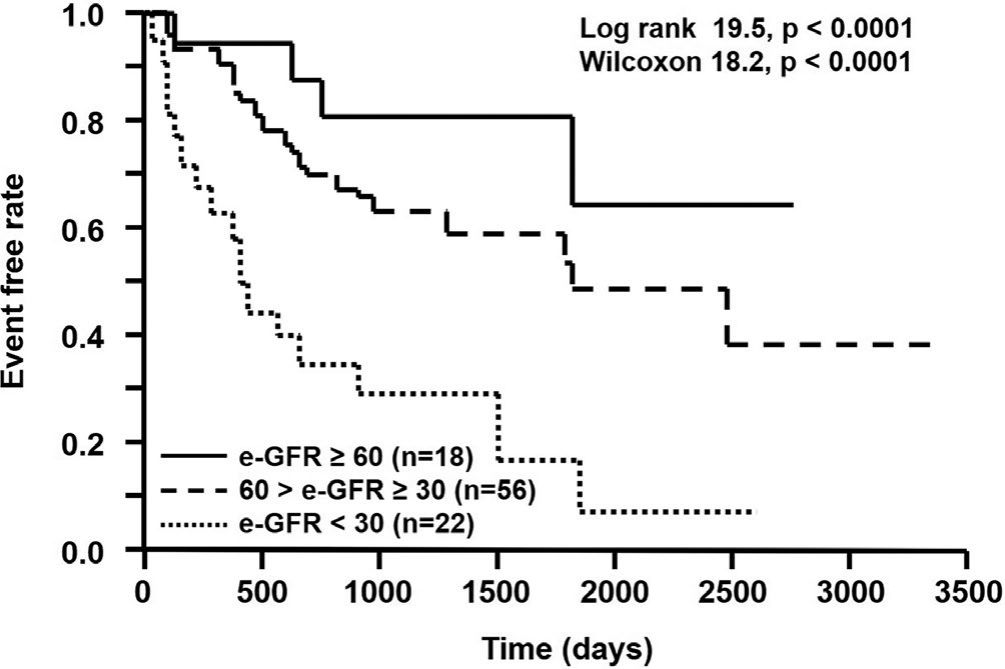
Kaplan‐Meier event‐free curves for MACCEs among three groups based on stages of CKD after judgment of CRT response. CKD, chronic kidney disease; CRT, cardiac resynchronization therapy; e‐GFR, estimated glomerular filtration rate (mL/min/1.73 m^2^), MACCEs, major adverse cerebral and cardiovascular events

Figure 2 showed the Kaplan‐Meier event‐free curves for MACCE among three groups based on stages of CKD after judgment of CRT response. MACCE‐free ratio was significantly lower depending on the depressed stage of renal function.

Table 2 showed the values of e‐GFR and BNP before CRT and immediately after the judgment of CRT response. BNP after the judgment of CRT response was greater in the nonresponders than in the responders. There was no significant change between the two groups.

**TABLE 2:** The chronological changes of e‐GFR and BNP according to the CRT response

	Nonresponders (n = 53)	Responders (n = 66)	*P* value
e‐GFR (ml/min/1.73 m^2^)			
before CRT	45 ± 17 (n = 53)	50 ± 23 (n = 66)	.24
after CRT	41 ± 19 (n = 39)	45 ± 18 (n = 58)	.31
Δ before CRT ‐ after CRT	−5.2 ± 1.8 (n = 39)	−4.5 ± 1.5 (n = 58)	.78
BNP (pg/mL)			
before CRT	569 ± 472 (n = 44)	431 ± 386 (n = 62)	.10
after CRT	421 ± 412 (n = 30)	210 ± 209 (n = 52)	.0029**
Δ before CRT ‐ after CRT	−108 ± 424 (n = 30)	−239 ± 370 (n = 51)	.15

Abbreviations: after CRT, after judgment of CRT response; before CRT, baseline value before CRT; BNP, brain natriuretic peptide; CRT, cardiac resynchronization therapy; e‐GFR, estimated glomerular filtration rate; Δ Before CRT ‐ after CRT, change of value between before CRT and after CRT.

** *P* < .01.

Table 3 showed the values of e‐GFR before CRT and after the judgment of CRT response. The e‐GFR before CRT and after the judgment of CRT response was lower, and BNP after judgment of CRT response was greater in patients with MACCEs than those without in responders. The baseline BNP before CRT, and that after judgment of CRT response were greater in patients with MACCEs than those without in nonresponders. There was no significant change of the other values for the occurrence of MACCEs between the two groups.

**TABLE 3:** The chronological changes of e‐GFR and BNP according to the occurrence of MACCE

	Nonresponders			Responders		
	with MACCE	without MACCE	*P* value	with MACCE	without MACCE	*P* value
e‐GFR (ml/min/1.73 m^2^)						
before CRT	43 ± 16 (n = 33)	49 ± 19 (n = 20)	.20	24 ± 38 (n = 24)	56 ± 24 (n = 42)	.002**
after CRT	36 ± 18 (n = 23)	47 ± 18 (n = 16)	.06	37 ± 17 (n = 23)	49 ± 18 (n = 35)	.01*
Δ before CRT ‐after CRT	‐6.7 ± 7.3 (n = 23)	‐3.0 ± 11 (n = 16)	.21	‐1.2 ± 7.7 (n = 23)	‐6.7 ± 15 (n = 35)	.10
BNP (pg/ml)						
before CRT	712 ± 500 (n = 30)	264 ± 186 (n = 14)	.002**	467 ± 391 (n = 23)	409 ± 386 (n = 39)	.57
after CRT	546 ± 470 (n = 19)	207 ± 117 (n = 11)	.03*	307 ± 172 (n = 20)	150 ± 210 (n = 32)	.007**
Δ before CRT ‐ after CRT	‐126 ± 508 (n = 19)	‐77 ± 235 (n = 11)	.77	‐205 ± 340 (n = 20)	‐260 ± 393 (n = 31)	.61

Abbreviations: after CRT, after judgment of CRT response; before CRT, baseline value before CRT; BNP, brain natriuretic peptide; CRT, cardiac resynchronization therapy; e‐GFR, estimated glomerular filtration rate; Δ Before CRT ‐ after CRT, change of value between before CRT and after CRT.

* *P* < .05,

** *P* < .01.

### 3.3 Difference of outcome by response to CRT

**FIGURE 3:**
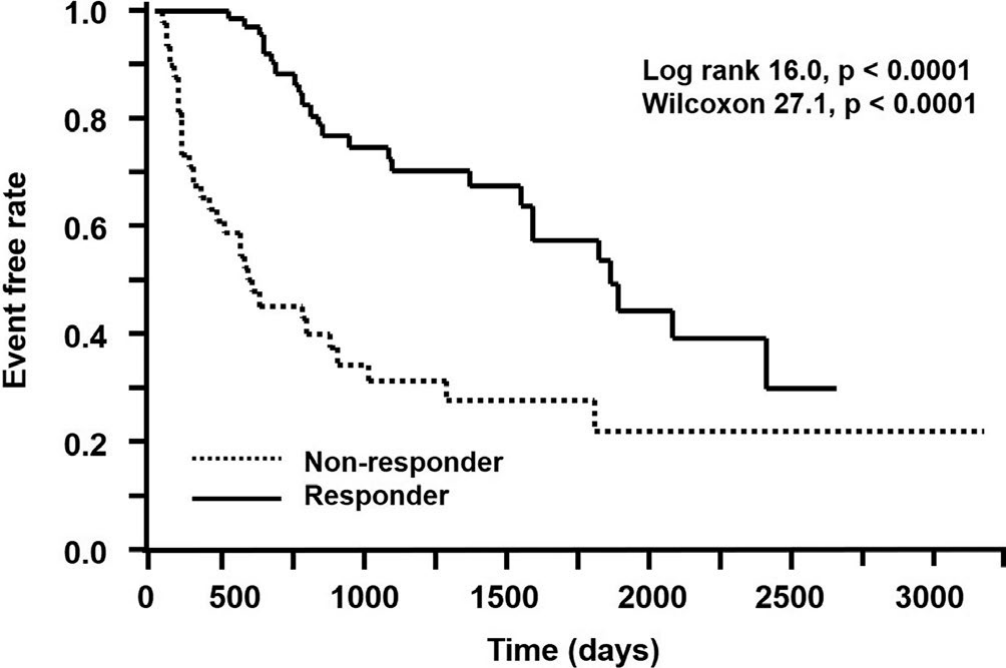
Kaplan‐Meier event‐free curves for MACCEs between the responders and nonresponders. MACCEs, major adverse cerebral and cardiovascular events

During the follow‐up period (706 ± 821 days for the nonresponders and 1207 ± 715 days for the responders), MACCEs were evident in 57 patients (48%). Specifically, 33 patients in the nonresponders developed MACCEs (congestive HF requiring admission = 30, nonfatal myocardial infarction = 1, and implantable cardioverter‐defibrillator treatment for fatal arrhythmia = 2), while only 24 patients in the responders developed MACCEs (congestive HF requiring admission = 19, nonfatal myocardial infarction = 1, implantable cardioverter‐defibrillator treatment for fatal arrhythmia = 1, and stroke = 3). The prevalence of MACCEs was significantly greater in the nonresponders than in the responders (62% vs 36%, *P* < .005). Significantly, the survival rate with MACCE‐free ratio, as assessed by Kaplan‐Meier analysis, was greater in the responders than in the nonresponders (log rank, 16.0; *P* < .0001; Wilcoxon, 27.1; *P* < .0001) (Figure 3). Approximately 1500 days after CRT, the number of MACCEs in the responders increased and approached the number of MACCEs in the nonresponders.

In the nonresponders, baseline plasma BNP before CRT was higher in patients with MACCEs than in patients without MACCEs (712 ± 500 pg/mL vs 264 ± 186 pg/mL; *P* < .005). In the responders, baseline e‐GFR before CRT was lower in patients with MACCEs than in patients without MACCEs (38 ± 16 mL/min/1.73 vs 56 ± 24 mL/min/1.73; *P* < .005).

Univariate Cox proportional hazards regression analysis revealed that the age and both levels of BNP before CRT and after judgment of CRT response were associated with MACCE in nonresponders, and both levels of e‐GFR before CRT and after judgment of CRT response and BNP after judgment of CRT response were associated with MACCEs in responders, as shown in Table 4.

**TABLE 4:** Univariate Cox proportional hazards regression analysis of the MACCE

	Nonresponders		Responders	
	Univariate *P* value	Hazard ratio (95% CI)	Univariate *P* value	Hazard ratio (95% CI)
Age (y)	.0309*	1.041 (1.174‐36.615)	.0558	1.047 (0.999‐1.103)
Gender (Female)	.578	0.811 (0.367‐1.666)	.239	1.620 (0.723‐3.693)
Dilated cardiomyopathy	.252	0.637 (0.268‐1.357)	.299	0.635 (0.268‐1.508)
QRS duration (ms)	.497	1.004 (0.992‐1.018)	.605	0.995 (0.978‐1.005)
SBP (mmHg)	.667	1.004 (0.984‐1.022)	.397	0.991 (0.969‐1.012)
DBP (mmHg)	.663	1.007 (0.973‐1.039)	.855	1.003 (0.968‐1.041)
Heart rate (beats/min)	.437	1.008 (0.988‐1.026)	.476	0.988 (0.956‐1.021)
LVEF (%)	.090	0.966 (0.928‐1.005)	.907	1.003 (0.955‐1.054)
End‐diastolic volume (ml)	.638	1.001 (0.995‐1.008)	.212	0.995 (0.987‐1.002)
End‐systolic volume (mL)	.348	1.004 (0.996‐1.011)	.279	0.995 (0.985‐1.004)
e‐GFR (mL/min/1.73 m^2^) before CRT	.247	0.988 (0.968‐1.008)	<.0001**	0.943 (0.911‐0.972)
e‐GFR (mL/min/1.73 m^2^) after CRT	.083	0.980 (0.957‐1.003)	.0004**	0.943 (0.915‐0.977)
BNP (pg/mL) before CRT	.0011**	1.001 (1.001‐1.002)	.380	1.000 (0.999‐1.001)
BNP (pg/mL) after CRT	.0053**	1.002 (1.001‐1.003)	.0021**	1.003 (1.001‐1.004)

Abbreviations: after CRT, after judgment of CRT response; before CRT, baseline value before CRT; BNP, brain natriuretic peptide; CRT, cardiac resynchronization therapy; DBP, diastolic blood pressure; e‐GFR, estimated glomerular filtration rate; LVEF, left ventricular ejection fraction; MACCE, major adverse cerebral and cardiovascular events; SBP, systolic blood pressure.

* *P* < .05,

** *P* < .01.

Multivariate Cox proportional hazards regression analysis clarified that high baseline plasma BNP levels both before CRT and after judgment of CRT response were independently related with the incidence of MACCEs in the nonresponders (Table 5; Model 1, 2), whereas low e‐GFR levels both before CRT and after judgment of CRT response had independent relationship to the occurrence of MACCEs in the responders (Table 5; Model 3, 4). High plasma BNP levels after judgment of CRT response were independently related to the incidence of MACCEs in the responders (Table 5; Model 5).

**TABLE 5:** Multivariate Cox proportional hazards regression analysis of the MACCE

Valuable	Multivariate *P* value		Hazard ratio	95% CI
Nonresponders				
Model 1				
Age (y)	.129	1.027		0.992‐1.067
LVEF (%)	.536	0.987		0.945‐1.030
BNP before CRT (pg/mL)	.003**	1.001		1.000‐1.002
Model 2				
Age (y)	.211	1.040		0.980‐1.112
LVEF (%)	.399	0.978		0.928‐1.030
BNP after CRT (pg/mL)	.002**	1.002		1.0007‐1.003
Responders				
Model 3				
Age (y)	.697	1.010		0.964‐1.065
e‐GFR before CRT (mL/min/1.73 m^2^)	.0001**	0.945		0.912‐0.975
Model 4				
Age (y)	.296	1.026		0.979‐1.084
e‐GFR after CRT (mL/min/1.73 m^2^)	.0009**	0.945		0.906‐0.980
Model 5				
Age (y)	.950	1.002		0.947‐1.065
BNP after CRT (pg/ml)	.003**	1.003		1.001‐1.004

Abbreeviations: after CRT, after judgment of CRT response; before CRT, baseline value before CRT; BNP, brain natriuretic peptide; CRT, cardiac resynchronization therapy; e‐GFR, estimated glomerular filtration rate; LVEF, left ventricular ejection fraction; MACCE, major adverse cerebral and cardiovascular events.

** *P* < .01.

### 3.4 Comparison of patients by e‐GFR in responders

**FIGURE 4:**
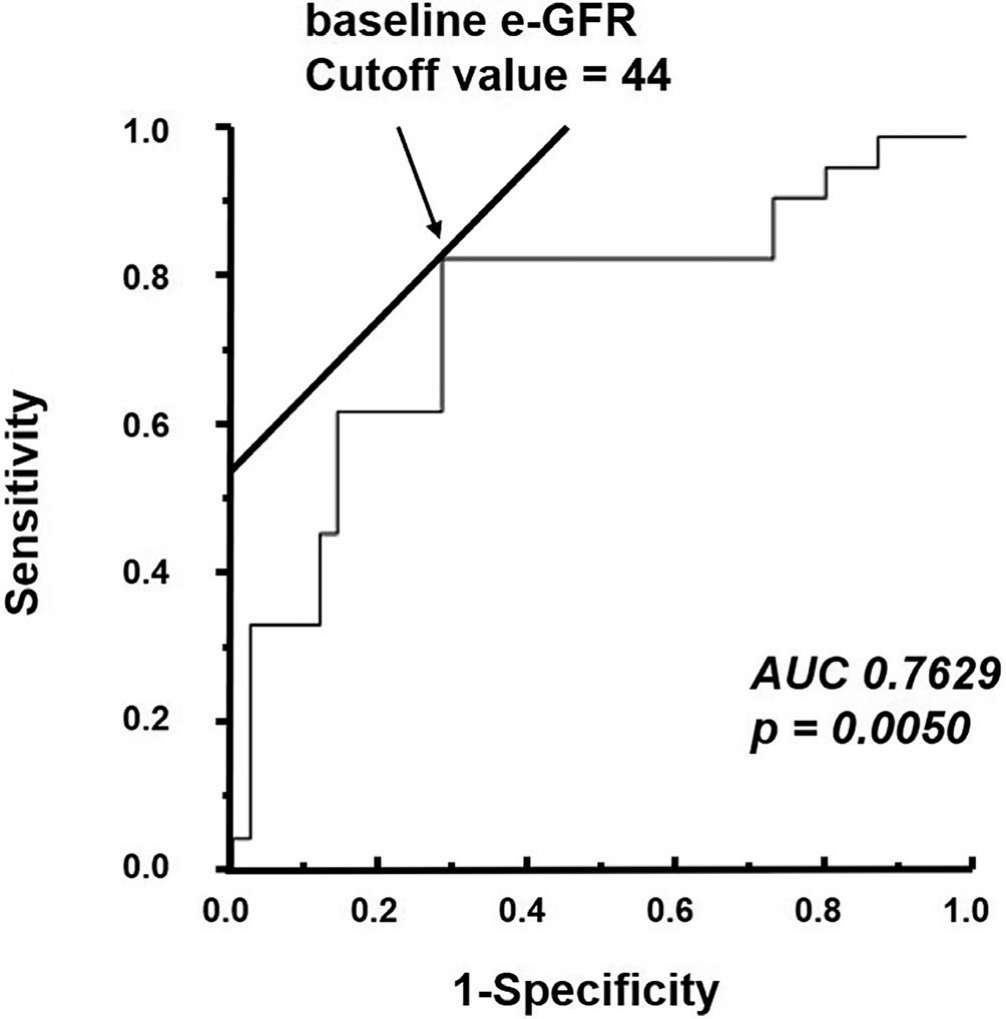
Receiver‐operating characteristic curve for the ability of baseline e‐GFR before CRT to predict MACCEs. AUC, area under the curve; CRT, cardiac resynchronization therapy; e‐GFR, estimated glomerular filtration rate; MACCEs, major adverse cerebral and cardiovascular events

On the basis of the receiver‐operating characteristic curve for predicting MACCEs, the baseline e‐GFR before CRT could be set at ≤44 mL/min/1.73 in responders (Figure 4). In the responders, Table 6 showed the baseline clinical characteristics between the preserved e‐GFR group and the depressed e‐GFR group. Patients in the depressed e‐GFR group were older than those in the preserved e‐GFR group (*P* < .05). The numbers of patients with cardiac sarcoidosis (*P* < .05) and baseline e‐GFR before CRT (*P* < .0001) were lower in the depressed e‐GFR group than the preserved e‐GFR group. There were no significant differences in gender, prevalence of atrial fibrillation, QRS duration, blood pressures, echocardiographic findings, and blood sample findings, except for e‐GFR.

**TABLE 6:** Baseline clinical characteristics of the patients according to baseline e‐GFR in responders

	Preserved e‐GFR	Depressed e‐GFR	*P* value
	(n = 34)	(n = 32)	
Age (y)	67 ± 9.7	72 ± 9.0	.012*
Gender (Female/male)	15/ 19	13/ 19	.77
Ischemic cardiomyopathy	3	7	.14
Dilated cardiomyopathy	20	16	.47
Cardiac sarcoidosis	8	1	.010*
Others	3	8	.07
Atrial fibrillation	4 (11.8%)	7 (21.9%)	.27
QRS duration (ms)	166 ± 25	171 ± 24	.36
Systolic blood pressure (mmHg)	112 ± 19	117 ± 18	.28
Diastolic blood pressure (mmHg)	65 ± 11	68 ± 11	.28
Heart rate (beats/min)	66 ± 13	65 ± 12	.68
LVEF (%)	28 ± 8.5	32 ± 8.0	.07
End‐diastolic volume (ml)	161 ± 59	163 ± 63	.86
End‐systolic volume (ml)	117 ± 51	115 ± 53	.85
e‐GFR before CRT (ml/min/1.73 m^2^)	65 ± 2.9	33 ± 3.0	<.0001**
BNP before CRT (pg/ml)	366 ± 328 n = 32	500 ± 434 n = 30	.18
Pro‐BNP before CRT (pg/ml)	1072 ± 841 n = 2	8043 ± 6556 n = 2	.85

Abbreviations: before CRT, baseline value before CRT; BNP, brain natriuretic peptide; CRT, cardiac resynchronization therapy; DBP, diastolic blood pressure; e‐GFR, estimated glomerular filtration rate; LVEF, left ventricular ejection fraction; SBP, systolic blood pressure.

* *P* < .05,

** *P* < .01

Through the follow‐up period (1001 ± 621 days in the depressed e‐GFR group and 1402 ± 750 days in the preserved e‐GFR group), MACCEs developed in 24 patients. In particular, 20 of the 34 patients in the depressed e‐GFR group developed MACCEs (hospitalization due to congestive HF, 16; nonfatal myocardial infarction, one; cardiovascular mortality, one; and stroke, two), whereas only four of the 34 patients in the preserved e‐GFR group developed MACCEs (HF requiring admission, three, and stroke, one). Thus, patients in the depressed e‐GFR group more frequently observed MACCEs than the patients in the preserved e‐GFR group (63% vs 12%; *P* < .0001).

### 3.5 Kaplan‐Meier MACCE‐free estimation between the groups by e‐GFR in responders

**FIGURE 5:**
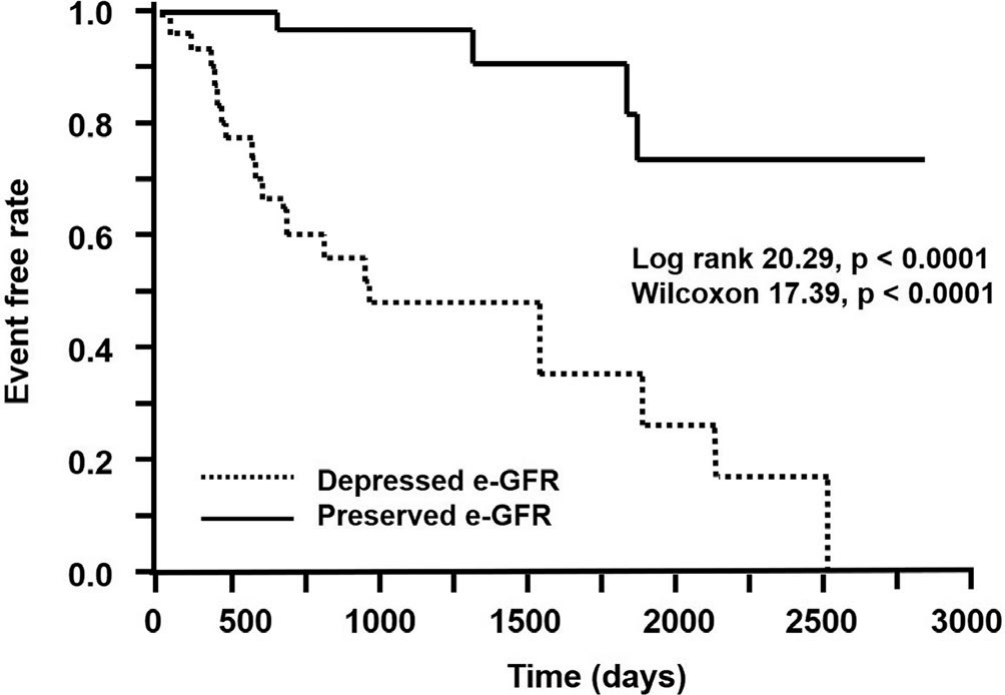
Kaplan‐Meier event‐free curves for MACCEs between patients with preserved e‐GFR and those with depressed e‐GFR in the responders. e‐GFR, estimated glomerular filtration rate; MACCEs, major adverse cerebral and cardiovascular events

In the responders, the MACCE‐free rate estimated by Kaplan‐Meier analysis was obtained that the patients in the preserved e‐GFR group were significantly higher than in the depressed e‐GFR group (log rank, 20.29; *P* < .0001 and Wilcoxon, 17.39; *P* < .0001) (Figure 5).

## 4 DISCUSSION

### 4.1 Main findings

In this study, MACCEs developed in 62% of the nonresponders and 36% of the responders. Approximately 1500 days after CRT, the number of MACCEs in the responders increased and approached the number of MACCEs in the nonresponders. The predictors of long‐term prognosis between the responders and nonresponders were distinctively different. The MACCE‐free ratio estimated by Kaplan‐Meier analysis was higher in the preserved e‐GFR group than in the depressed e‐GFR group. It was very crucial that the depressed e‐GFR exactly predicted MACCEs through the follow‐up period only in the responders.

### 4.2 Difference of risk factors for MACCEs by response to CRT

In this study, the risk factors for MACCEs were distinctively different between the responders and nonresponders through the follow‐up duration. In the nonresponders, baseline plasma BNP levels were associated with MACCEs. Alternatively, in the responders, e‐GFR levels were associated with MACCEs. The reason for the dissociation between the two groups was unclear. There were several varied disfavorable circumstance in the nonresponders, which included big fibrous scar, the lack of detected dyssynchrony by echocardiograph above device implantation, nonimprovement of dyssynchrony after device implantation, unsuitable position of pacing leads, and unsuitable program of the device. The observation in this study that the risk factors for MACCEs did not match between the responders and nonresponders may be elucidated by many different backgrounds of the patients in this study in each group.

CRT response rate in this study tended to be lower than that in the previous studies.^9^, ^12^ Baseline e‐GFR before CRT implantation was relatively low in all patients in this study; average e‐GFR: 45 mL/min/1.73 m^2^ (nonresponder) and 50 mL/min/1.73 m^2^ (responders), respectively. Impairment of renal function (e‐GFR <60 mL/min/1.73 m^2^) was directly associated with a significant increase in mortality.^13^ One of the reasons for low CRT response rate might be due to relatively lower baseline e‐GFR in our patients.

### 4.3 Renal dysfunction as a predicting factor for MACCEs in the responders

A study showed that many patients with HF after CRT had cardiorenal syndrome, a condition characterized by volume overload due to cardiac and renal dysfunctions. Patients with cardiorenal syndrome were more likely to experience MACCEs and become nonresponders with poor cardiac function improvement.^14^ Multiple risk factors cause the progression of cardiac and renal dysfunctions, and they lead to severe HF. Additionally, the interconnection between multiple organs permits multiple dysfunction in multiple interconnected organs, such as increasing sodium ion, holding plasma volume, and systemic inflammation, through the stimulation of the renin angiotensin aldosterone system and the neurohormonal system.^7^, ^15^ These multiple functional changes occur the cardiac remodeling and the glomerular filtration progression. As a result, both the kidneys and the heart are steadily impaired.^14^ Our results suggest that even in CRT responders judged 6 months after implantation, renal dysfunction at implantation requires close attention because of the potential high risk of developing MACCEs.

### 4.4 Long‐term outcomes in the responders

In a study, about one‐fifth of the patients after CRT became super responders with normalization of LVEF.^16^ Dramatic reverse LV remodeling is needed to obtain clinical improvement after 6 months of CRT and better survival and less hospitalizations for HF even after that.^16^ Alternatively, MADIT‐CRT, in Multicenter Automatic Defibrillator Implantation Trial with CRT, deaths or ICD activation for fatal arrhythmia developed in 12% of the responders approximately 3 years after CRT.^17^ Several responders revealed a response for only short term, and reverse remodeling effects of LV were transitory in part of the responders.^18^ They were called brief responders,^18^ transient responders,^6^ or late nonresponders.^19^ Brief responders demonstrate reverse LV remodeling in the short term. However, the beneficial effect could not be sustained through long period,^18^ and 35% of the responders developed MACCEs about 1200 days from undergoing cardiac resynchronization in their observation. Oka et al^18^ advocated that the mechanism of the transient effect was due to fewer viable myocardia, progression of the original disease, inadequate resynchronization, and other origins of contractive dysfunction without dyssynchrony. Ichibori et al showed that the response for CRT in about 33% of responders was temporary 2 years after operation. Transient responders had unfortunate outcomes over 7.6 year's follow‐up.^6^ Transient responders were determined by chronic atrial fibrillation and administration of amiodarone.^6^ However, unlike our study, renal dysfunction did not affect the prognosis of the responders in the aforementioned two studies on transient responders. The reason for the dissociation of the results is unclear, but it may be due to the differences in populations or analysis methods. Responders judged 6 months after CRT might need to undergo long‐term observations, considering the possibility of them being brief responders.

We should consider some limitations in this study. First, we estimated a small number of patients in a single center that might make the data analysis hard. Thus, future larger size studies are needed to detect the accurately outcome of the responders to CRT. Second, our analysis in this study was retrospective in nature, thus sampling bias and incomplete data might occurred. Consequently, prospective studies are necessary to detect the prognosis of the responders to CRT in future.

## 5 CONCLUSION

Our results suggest that the factors for MACCEs during a long follow‐up period were distinctively different between the responders and nonresponders who underwent CRT. We believe that patients with depressed e‐GFR have poor prognoses, even if they are CRT responders.

## CONFLICT OF INTEREST

The authors have no conflict of interest. This study was approved by the institutional review board (IRB) of Oita University, and the IRB approval number was B11‐053.
